# Case Series: COVID-19 in African American Patients With Sarcoidosis

**DOI:** 10.3389/fmed.2020.588527

**Published:** 2020-11-04

**Authors:** Michael Manansala, Christian Ascoli, Ana Goico Alburquerque, David Perkins, Mehdi Mirsaedi, Patricia Finn, Nadera J. Sweiss

**Affiliations:** ^1^Department of Internal Medicine, University of Illinois at Chicago, Chicago, IL, United States; ^2^Miller School of Medicine, University of Miami, Miami, FL, United States

**Keywords:** sarcoidosis, COVID- 19, immunosuppression, DMARDs, African American (AA), SAR-CoV-2

## Abstract

Data on the clinical presentation and outcomes of sarcoidosis patients with coronavirus disease 19 (COVID-19) are scarce. In this case series, we identified 5 out of 238 sarcoidosis patients who are enrolled in an ongoing longitudinal observational study who developed COVID-19 during the study period and follow their clinical course. Four patients recovered completely, whereas one patient expired during hospital admission. Our preliminary experience suggests that African American patients with chronic sarcoidosis treated with disease-modifying anti-rheumatic drugs (DMARDs) or anti-tumor necrosis factor (TNF) therapy do not seem to be at increased risk of respiratory or life-threatening complications from severe acute respiratory syndrome coronavirus 2 (SARS-CoV-2) compared with the general population, although at the present time, we advocate for maintaining a high level of vigilance and strict follow-up in this patient population.

## Introduction

The recent outbreak of severe acute respiratory syndrome coronavirus 2 (SARS-CoV-2) responsible for severe acute respiratory syndrome (SARS) represents a source of concern for the management of patients with sarcoidosis (1). Within the realm of sarcoidosis, it has been shown that African Americans have 12 times the rate of age-adjusted mortality compared with similar Caucasian patients (2). This is compounded by the observation that African American patients are at disproportionately increased risk of mortality and morbidity from coronavirus disease 19 (COVID-19) (3). Data on COVID-19 in patients with sarcoidosis are scarce. Additionally, there is a concern that immunocompromised patients are at increased risk of mortality from COVID-19. Here, we report a case series describing the clinical course of five African American patients with sarcoidosis after infection with SARS-CoV-2.

## Methods

We assessed patients with sarcoidosis care established at the University of Illinois of Chicago Bernie Mac STAR Clinic who are enrolled in an ongoing longitudinal observational study for SARS-CoV-2 infection during the period of March 12 to April 30, 2020. We identified five out of 238 patients (2.1%) with confirmed SARS-CoV-2 infection by PCR and clinical symptoms consistent with COVID-19 disease. Demographic and clinical data were collected.

## Case Presentation

We identified 5 out of 238 sarcoidosis patients who were infected with SARS-Cov-2 during the study period. All patients were of African American descent. The most common presenting symptom was cough. One patient had an atypical presentation of gastrointestinal discomfort and diarrhea. Four patients recovered completely despite having comorbidities and being on chronic immunosuppression. Two of the five patients did not receive any additional treatment for COVID-19. Three of the five patients received hydroxychloroquine (HCQ) and azithromycin for treatment for COVID-19. No changes were made to the patients' current immunosuppressive regimen. They also did not experience significant relapses of sarcoidosis from the time of COVID-19 diagnosis to date. One patient died after developing a likely thromboembolic event during hospitalization in the intensive care unit (ICU). Additional clinical characteristics of these patients are summarized in Table 1. The clinical data, including symptoms, laboratory data, and outcomes, are included in Table 2.

**Table 1:** Clinical characteristics of five African American sarcoidosis patients with confirmed COVID-19.

	**Patient 1**	**Patient 2**	**Patient 3**	**Patient 4**	**Patient 5**
Age	45	62	50	48	46
Gender	M	F	M	F	M
Sarcoidosis clinical phenotype requiring treatment	Pulmonary	Advanced pulmonary	Ocular cardiac	Neurologic	Testicular
BMI	28	28	31	46	43
Comorbidities	Asthma	Pulmonary hypertension	Uncontrolled hypertension, uncontrolled diabetes	Uncontrolled hypertension	Uncontrolled hypertension
Smoking history	None	None	Active smoker	None	Active smoker
Sarcoid medications at the time of presentation	Methylprednisolone 8 mg daily	MTX 10 mg weekly, HCQ 200 mg daily, methylprednisolone 4 mg daily	None	None	Infliximab every 8 weeks, MTX 7.5 mg weekly

*BMI, body mass index; MTX, methotrexate; HCQ, hydroxychloroquine*.

**Table 2:** Clinical data for five African American sarcoidosis patients with confirmed COVID-19.

	**Patient 1**	**Patient 2**	**Patient 3**	**Patient 4**	**Patient 5**
COVID-19 symptoms	Dyspnea on exertion, cough	Cough, anosmia, dysgeusia, myalgia	Cough, shortness of breath, fever, myalgias, diarrhea	Cough, fever	Diarrhea
Symptom duration prior to presentation (days)	2	7	4	7	10
**Relevant laboratories prior to presentation**					
WBC (3.9–12 thou/μl)	3.8	7.2	3.9	6.7	3.9
Abs lymphocytes (1.3–4.2 thou/μl)	1.7	1.7	1.3	1.9	1.8
Abs CD4 (438–1,501 cells/mm^3^)	696	1,107	–	1,501	833
CRP (0–18 mg/L)	<1.0	6.9	6.8	4.3	4.9
ESR (0–10 mm/h)	8	19	16	64	21
**Relevant laboratories at the time of**
**presentation**		
WBC (3.9–12 thou/μl)	4.5	–	3.1	–	2.8
Abs lymphocytes (1.3–4.2 thou/μl)	1.4	–	0.9	–	1.2
Abs CD4 (438–1,501 cells/mm^3^)	–	–	152	–	420
LDH (90–180 U/L)	273	–	270	–	–
Ferritin (10–259 ng/ml)	152	–	730	–	–
CRP (0–18 mg/L)	1	–	62.5	–	10.3
ESR (0–10 mm/h)	–	–	40	–	55
COVID-19 treatment	None	HCQ, azithromycin	HCQ, azithromycin, hydrocortisone, tocilizumab	HCQ, azithromycin, prednisone	None
Chest X-ray at the initial COVID-19 evaluation	No acute cardiopulmonary process	Diffuse advanced interstitial lung disease	Bibasilar reticular infiltrates, later progressed to bilateral pulmonary opacities consistent with ARDS	Bibasilar atelectasis, bilateral pulmonary opacities	No acute cardiopulmonary process
Outcome	Discharged to home from ED, doing well at 2-week follow-up	Did not require hospitalization, complete recovery at 2-week follow-up	Death due to pulmonary embolism after prolonged hospitalization requiring ICU admission	Discharged from the ICU, doing well at 2-week follow-up	Did not require hospitalization, complete recovery at 2-week follow-up

*Available relevant laboratory data, treatment, and outcome at follow-up are presented*.

*WBC, white blood cell; Abs, absolute; CRP, C-reactive protein; ESR, erythrocyte sedimentation rate; LDH, lactate dehydrogenase; ED, emergency department; HCQ, hydroxychloroquine; ARDS, acute respiratory distress syndrome; ICU, intensive care unit*.

## Discussion

The case fatality rate of COVID-19 is estimated to be 1–6% in the general population (4). Currently, the Center for Disease Control and Prevention (CDC) lists several risk factors for severe COVID-19, including immunocompromised status (5). However, preliminary data from an observational study of 320 Italian patients on immunosuppressive therapy for rheumatoid arthritis did not show increased risk of respiratory or life-threatening complications from SARS-CoV-2 compared with the general population (6). Indeed, it has been postulated that the pathogenesis of severe COVID-19 disease is in large part due to virally driven hyperinflammation that is perpetuated by the host immune response (7–9). Analysis from a cohort of COVID-19 cases from Wuhan, China revealed that patients requiring ICU had increased levels of pro-inflammatory cytokines (10).

Our findings do not provide any conclusions on the incidence rate of SARS-CoV-2 infection in patients with sarcoidosis, nor on the severity or overall outcome of immunocompromised sarcoidosis patients affected by COVID-19 disease. However, our preliminary experience suggests that African American patients with chronic sarcoidosis treated with disease-modifying anti-rheumatic drugs (DMARDs) or anti-tumor necrosis factor (TNF) therapy do not seem to be at increased risk of respiratory or life-threatening complications from SARS-CoV-2 compared with the general population. Better understanding of the implications of COVID-19 in patients with sarcoidosis and the effects of immunosuppressive therapies on COVID-19 infection outcome is urgently needed to guide clinicians in patient care. At the present time, we advocate for maintaining a high level of vigilance and strict follow-up in this patient population, including the exclusion of superimposed infections.

## Data Availability Statement

The raw data supporting the conclusions of this article will be made available by the authors, without undue reservation.

## Ethics Statement

Written informed consent was obtained from the individual(s) for the publication of any potentially identifiable images or data included in this article.

## Author Contributions

NS identified patients of interest within the cohort. MMa wrote the manuscript with assistance from CA, AA, DP, PF, and MMi. All authors contributed to the article and approved the submitted version.

## Conflict of Interest

The authors declare that the research was conducted in the absence of any commercial or financial relationships that could be construed as a potential conflict of interest.
